# Temporal Trends in Mortality from Alzheimer’s Disease in Federal District, Brazil: An Ecological Study (2010–2018)

**DOI:** 10.3390/ijerph20186713

**Published:** 2023-09-05

**Authors:** Sarah dos Santos Conceição, Delmason Soares Barbosa de Carvalho, Josicélia Estrela Tuy Batista, Amanda Oliveira Lyrio, Elivan Silva Souza, Paulo José dos Santos de Matos, Alexandre Marcelo Hintz, Simone Seixas da Cruz, Isaac Suzart Gomes-Filho, Ana Claudia Morais Godoy Figueiredo

**Affiliations:** 1 Department of Health, University of Brasília, Brasília 70910-900, Brazil; 2 Health Surveillance, Department of Public Health of the Federal District, Brasília 70390-125, Brazil; 3 Department of Health, Feira de Santana State University, Feira de Santana 44036-900, Bahia, Brazil; 4 Department of Epidemiology, Federal University of Recôncavo of Bahia, Santo Antônio de Jesus 44574-490, Bahia, Brazil

**Keywords:** Alzheimer disease, dementia, aged, mortality

## Abstract

Introduction: Neuropsychiatric diseases, particularly dementias, has become more prominent with a great impact on the quality of life of the elderly population. Objective: To verify the rate of increase in mortality due to Alzheimer’s disease in the Federal District, Brazil from 2010 to 2018. Method: An ecological study was conducted, with a time series, about the evolution of the mortality coefficient in the Federal District, Brazil carried out at the Federal District State Department of Health. Mortality rates were defined as the dependent variable and years evaluated as the independent variable—from 2010 to 2018. For temporal trend analysis, the Prais–Winsten linear regression model was used and the increment rate with the respective 95% confidence interval was estimated. Results: From 2010 to 2018, 1665 deaths which had Alzheimer’s disease as the underlying cause were recorded in the Mortality Information System. The results showed an overall mortality rate of 6.55 deaths per 100,000 inhabitants, with a higher predominance in females, non-Black people, and those aged 80 years or older. There was an increase in the annual trend of the overall mortality coefficient in both sexes. Conclusion: The findings demonstrated a significant increase in the temporal evolution of mortality due to Alzheimer’s disease in the Federal District, Brazil. It was recommended to conduct original studies to evaluate the factors that can cause the disease in order to collaborate in the process of formulating policies in the area of public health and improvements in clinical practice.

## 1. Introduction

The rapid aging of the Brazilian population due to demographic transition has produced significant changes in the country’s age pyramid [1]. Simultaneously with this process, there have been changes in morbidity and mortality patterns, with the ascendancy of chronic degenerative diseases. Neuropsychiatric diseases, particularly dementias, have become more frequent with a great impact on the quality of life of the elderly population, resulting in loss of autonomy, memory, and cognitive deterioration, in addition to consequences for mental and social integrity and the ability to function, becoming a relevant public health problem [2,3,4,5].

Worldwide, about 50 million people are diagnosed with dementia, and the prevalence shows variability from 4.7% to 8.7% according to the continent data [3,6]. In 2010, there were 35.6 million people with dementia, but 65.7 million individuals are expected to be affected by the outcome by 2030 and 115.4 million by 2050. Each year, approximately 7.7 million new cases appear, which means that one person is diagnosed every four seconds [7,8].

Since 1990, the prevalence, morbidity and mortality attributable to the occurrence in Brazil have increased by 2.5, 3.4 and 2.3 times, respectively. This rate of growth led the country to the second highest prevalence of assisted persons standardized by age in the world in 2016. With the current demographic transition and the aging of the population aged 60 or over, Brazil will face a fourfold increase in the number of people living with the disease from approximately 927,000 in 2010 to 3,728,000 in 2050.

The etiology of dementia, however, is not clearly established [2,3,4,5]. Among the various situations that can cause the disease are the process of degeneration of nerve cells in the brain, the use of toxic substances such as alcohol or drugs, nutritional deficiencies, infections, some types of hydrocephalus and brain trauma. Increased age and genetic aspects are considered important risk factors for the occurrence of dementia. This disease can be stratified into several types such as vascular, Parkinson’s, mixed dementia and Alzheimer’s [9].

Alzheimer’s disease, a type of dementia which is the focus of the present study, is the most frequent neurodegenerative dysfunction related to advancing age, the cognitive manifestations of which result in progressive deficiency and eventual disability [10]. It is estimated that the number of people with Alzheimer’s disease will double every 20 years [4,11].

Different countries have reported a fall in mortality rates from chronic diseases. However, the trend of mortality due to Alzheimer’s disease increased considerably in both sexes and in the age groups of over 60 years [12]. In 2018, Alzheimer’s was the fifth leading cause of death worldwide. In Brazil, projections revealed that the prevalence of the disease was higher than that observed worldwide. In the population aged 65 years or older, this prevalence increased from 7.6% to 7.9% between 2010 and 2020, i.e., 55,000 new cases per year [13].

Studies on mortality due to Alzheimer’s disease are scarce and conflicting in Brazil. Most investigations address dementia in general or are limited to a small sample size, usually in hospital units, with institutionalized individuals or socioeconomically differentiated groups [4,14]. Studies on the topic are important to elucidate aspects related to public health and the clinical practice of health professionals. In this context, due to lack of knowledge on the topic, the present study aimed to verify the temporal trend of mortality due to Alzheimer’s disease in the Federal District, Brazil, from 2010 to 2018.

## 2. Method

### 2.1. Design and Context of the Research

We conducted a time series study of the evolution of the mortality rate due to Alzheimer’s disease in the Federal District (DF), Brazil, in a period from 2010 to 2018. The choice of the period of investigation was due to the fact that the best quality of data was achieved from 2010 onwards, when, through the comparison of the individual clinical histories, the confirmation of the diagnosis of Alzheimer’s disease was carried out. The period from 2010 to 2018 was chosen because at the time of the information survey, the data available for evaluation only existed until the year 2018. This federative unit has a territorial area of 5,760,783 km², divided into 33 administrative regions. In 2018, the estimated number of inhabitants was 2,974,703 [15], and 12,157 individuals died [15]. The Federal District Department of Health has a trained multidisciplinary team to verify the quality of the data available in the Mortality Information System and the information contained in a death certificate. The Research Ethics Committee of the Health Sciences Teaching and Research Foundation approved the development of the study (CAAE protocol: 95486818.0.0000.5553).

### 2.2. Target Population and Eligibility Criteria

The study population was composed of resident individuals who died between 1 January 2010 and 31 December 2018 and who presented Alzheimer’s disease as the underlying cause of death. Deaths classified according to codes G30.0, G30.1, G30.8 and G30.9 of the 10th version of the International Classification of Disease—ICD 10 were included in the study. Inconclusive cases or clinical histories of people who died with incomplete data, as well as individuals diagnosed with other dementias, were excluded.

### 2.3. Source and Data Collection Instruments

The Mortality Information System includes national data and allows the consultation of individual data from each death certificate. For the present study, the regional system of the Federal District was consulted. Within the Federal District, since 2010, the Department of Health, together with all hospital units in the State, made efforts to qualify the information recorded in death certificates. For this purpose, a previously trained physician from the secretariat (A.C.M. or D.C.B.S) conducted the in loco assessment of the medical record of the individual who died in the hospital unit, filling out a standardized document. This document presents information related to the clinical history of the disease, such as the period that presented the initial symptoms, clinical diagnosis of Alzheimer’s disease by a neurologist or geriatrician, ands image examination, among others.

### 2.4. Investigated Variables

Mortality rates were defined as a dependent variable and the years of occurrence of deaths were defined as an independent variable. For the characterization of the population, the following covariates were verified and grouped into categories: age in years (45 to 59; 60 to 69; 70 to 79 and 80 or more), race/skin color (black and non-black), sex (female and male), schooling level in years of study (none, 1–3 years, 4–7 years, 8–11 years and greater or equal to 12 years), marital status (married/stable union and single/divorced/widowed) and region of residence.

### 2.5. Procedures for Data Collection

Initially, the screening of all death certificates was conducted to verify the underlying cause of death filled in, classifying them as well or poorly defined. The latter were designated for further investigation by previously trained staff. The investigation consisted in the evaluation of the clinical history of people who died using documents made available by the Federal District Department of Health, from which the database was constructed. The most recent population estimates were taken from the website of the Planning Company of the Federal District [15], which presents the inter-census numbers of the population according to age group, sex and administrative region.

### 2.6. Procedures for Data Analysis

The database was built for analysis in the STATA (Data Analysis and Statistical Software) program, version 16, serial number 301606315062. Descriptive analysis was performed for all categorical variables according to the relative and absolute frequencies. Subsequently, mortality rates per 100,000 people were calculated, that is, the number of deaths from Alzheimer’s disease divided by the total number of residents in the Federal District. In the temporal trend analysis, the Prais–Winsten linear regression model, using the increment rate and a 95% confidence interval, quantified the annual variations of mortality indicators. The rates were considered constant when the regression coefficient was not statistically significant, increased when there was positive significance, and decreased when there was negative significance, according to confidence intervals.

## 3. Results

A total of 1665 deaths due to Alzheimer’s disease in the population living in the Federal District, Brazil were recorded in the Mortality Information System. Most of the patients were 80 years or older, women, non-Black, without a partner and had 1 to 3 years of schooling. The highest incidence of deaths (18.55%) occurred in 2017 (Table 1).

There was an average annual growth of 33.40% in mortality due to Alzheimer’s disease as the underlying cause of death between 2010 and 2018. Among men, the increase was 31.42%, and in females there was an increase of 36.88%. Regarding the age groups analyzed, the increasing temporal evolution of mortality in individuals aged 80 years or more stands out, with an increase of 23.1%. However, a constant trend was observed in individuals under 80 years of age (Table 2).

**Figure 1:**
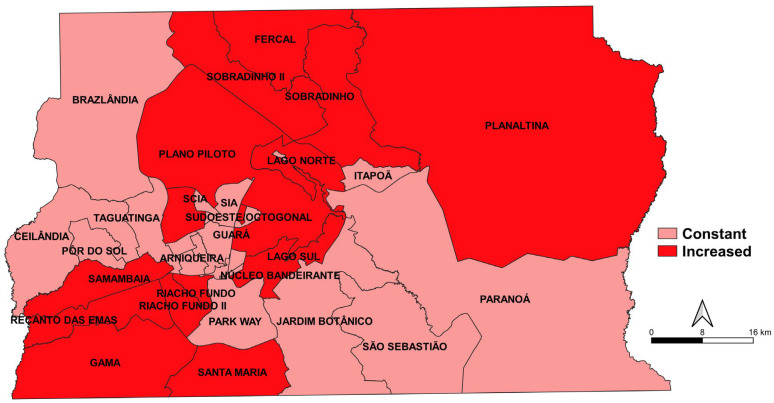
Rate of increase in mortality from Alzheimer’s disease, according to the Administrative Region of the Federal District, Brazil, 2010 to 2018.

The rate of increase in mortality due to Alzheimer’s disease for each Administrative Region was verified separately (Figure 1). Growth was more pronounced in some regions, with an increasing trend of 82.97%, 75.02% and 70.31%, respectively, in Samambaia, Planaltina and Santa Maria (Table S1 of Supplementary Materials).

**Figure 2:**
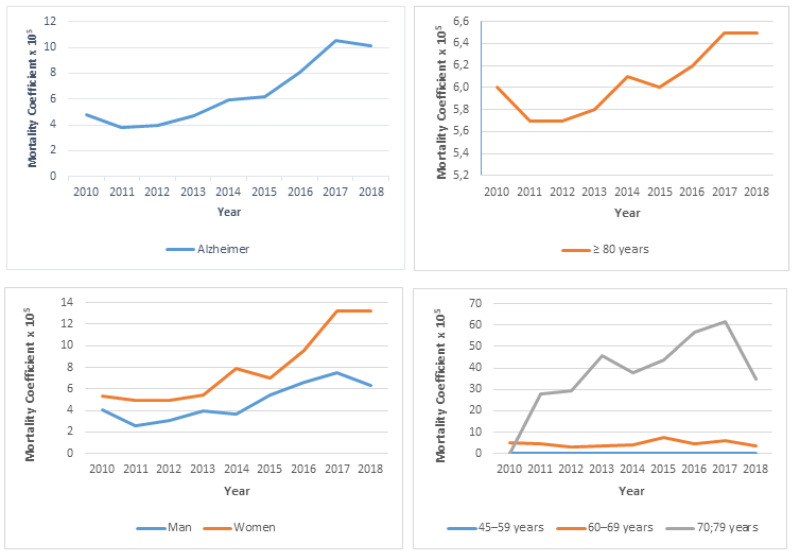
Mortality rate due to Alzheimer’s disease in the Federal District per 100,000 inhabitants, according to gender and age group, between 2010 and 2018.

The mean mortality rate due to Alzheimer’s disease was 6.55 per 100,000 inhabitants. There was a gradual increase in the incidence of deaths, with the years 2017 and 2018 presenting the highest values for the mortality coefficient (Figure 2). In females, this indicator was 8.11, while in men it was 4.86. The age group of 80 years or older had a higher proportion of deaths, representing a rate of 56.28 deaths per 100,000 individuals (Table S2 of Supplementary Materials).

Considering the most recent population estimates, the data regarding the administrative regions (Table S3 Supplementary Materials) showed that the regions of Lago Sul, Lago Norte, Plano Piloto and Cruzeiro were responsible for the highest mortality rate means, presenting coefficients of 25.22; 18.80; 15.41 and 15.27 per 100,000 inhabitants, respectively (Table 3).

## 4. Discussion

The results of the present study showed an increasing trend in the overall mortality coefficient and in both sexes, with a higher predominance in females, individuals with non-Black complexion and aged 80 years or older. In this context, it was ratified that Brazil is undergoing a process of population aging, providing greater life expectancy and, consequently, an increase in dementia diseases [4,16,17].

The growth pattern of mortality from Alzheimer’s disease can also be verified worldwide [18]. In the United States of America, a previous study [19] revealed an increase in the temporal evolution of Alzheimer’s disease mortality of 43%. According to another North American study [20] conducted from 2000 to 2008, Alzheimer’s disease had an exponential increase of 66%.

In China, between 2009 and 2015, there was an increase in the death rate from Alzheimer’s disease by sex and age [21]. In Latin American countries, such as Venezuela, a study conducted from 1988 to 1998 [22] revealed an increase in the mortality rate due to Alzheimer’s disease from 0.22 to 5.5/100,000 inhabitants. Among men, it increased from 0 to 4.7/100,000 inhabitants, and in relation to age groups, the mortality rate was higher in females over 75 years of age, corroborating the results of the present study.

Recent scientific evidence points out that Alzheimer’s disease disproportionately affects women compared to men, which can be a consequence of genetic sexual alterations and the predominance of females in the elderly population [23]. Moreover, research conducted in Australia [24] indicates that the process of cerebral atrophy, cognitive decline and clinical progression of the disease is more accelerated in females, whose biological mechanism is based on the theory that hormonal changes, with decreased estrogen levels after menopause, can potentiate changes caused by the disease.

A prospective study [25] conducted in Italy with the objective of evaluating the factors associated with differences in the evolution of and mortality due to Alzheimer’s disease among males and females found that men had more comorbidities, but women showed a greater functional decline. In the Brazilian reality, research conducted in the country’s capitals (except Brasília) between 2000 and 2009 pointed out that women had a higher risk of triggering Alzheimer’s disease in the most advanced stages of life due to their greater survival rate, justifying the higher mortality from the disease in females, especially over 80 years old [4].

Regarding complexion, previous studies have shown that Black people presented a lower proportion of the occurrence of the disease, which may have a possible justification in the fact that there are differences in genetic biomarkers of this population favorable to neurocognitive preservation, corroborating the findings of the present study [26]. In addition to genetic factors, the lower life expectancy of this population may also justify the different rates when compared to non-Black people [10,27]. Previous American research conducted in the city of Chicago also found a faster cognitive decline in non-Black when compared to Black people [28].

The present investigation still confirmed the highest mortality rates due to Alzheimer’s among the elderly and less educated people [8]. Higher rates of deaths from Alzheimer’s were concentrated in poorer regions in Brazil [29], demonstrating that socioeconomic status has an influence on illness. However, in the present investigation, when analyzing the mortality trend by administrative region of the Federal District, Brazil, an increasing pattern was observed in most administrative regions. It was observed that regions with worse socioeconomic conditions, such as Samambaia, Santa Maria and Planaltina, presented higher rates of increase in mortality when compared to other locations. A possible justification for this result may be the early diagnosis of Alzheimer’s disease in the studied population.

In this scenario, population-based studies are an important tool to estimate indicators of health-related conditions and behaviors. In recent decades, there has been increasing interest in this type of study, as it generally denotes the predictive measurement capacity in adult mortality and functional capacity of the elderly [30]. For this purpose, the population confidence interval was estimated, with the intention of verifying the variability of the estimated measurement. Due to the nature of the study, it was not possible to rule out the possibility of underreporting, and this needs to be assumed to be a limitation. Furthermore, the low frequency of deaths in some of the specific variable categories can lead to spurious interpretations.

Among the strengths of this research was the fact that we used the total cases of death from Alzheimer’s disease of the population living in the Federal District, Brazil identified in the studied period. It is noteworthy that all cases were evaluated by specialized and previously trained health professionals in order to reduce the possibility of selection and information biases. The data obtained on mortality are representative of the Federal District, Brazil for the years 2010 to 2018; however, it is necessary to be careful when applying the precepts of generalization to other locations and populations.

Despite the coherence found between this study and others conducted at national and international levels, it is worth highlighting some limitations. Due to the characteristic inherent to ecological studies, it was not possible to make causal inference, and estimates of the ecological effect should not be expected to denote information at the individual level. Another major factor was the completeness of the filling of the death certificates, especially in the elderly population, since they usually present several comorbidities, which can generate underreporting, especially in relation to chronic degenerative diseases.

## 5. Conclusions

The present study demonstrated a significant growing trend in overall mortality in both sexes due to Alzheimer’s disease. Due to few existing studies on the topic at the national level, the study design strategy, which was aimed at analyzing all deaths with Alzheimer’s disease as the underlying cause, captured the magnitude of the disease more intensely. We hope to have contributed to the process of formulating policies in public health for the elderly living in the Federal District, Brazil for care practices of the teams that assist this population, and to comparing data on the topic at an international level. Lastly, it is recommended that original studies should be conducted to evaluate the factors that may cause this disease. Brazilian researchers interested in Alzheimer’s disease need to focus more on original studies to put into practice public policies for the groups most exposed to the outcome.

## Figures and Tables

**Table 1:** Sociodemographic characteristics of the population that died of Alzheimer’s disease in the Federal District, Brazil, from 2010 to 2018 (*n* = 1655).

Characteristics	N	%
Age (Years) *^100^		
45–59	5	0.30
60–69	65	3.93
70–79	204	18.37
≥80	1281	77.40
Sex		
Male	589	35.59
Female	1066	64.41
Schooling level (years of study) *^169^		
None	283	19.04
1–3	488	32.84
4–7	220	14.80
8–11	273	18.37
≥12	222	14.95
Marital status *^45^		
Without a partner	1074	66.71
With a partner	536	33.29
Race/complexion *^29^		
Non-Black	1164	71.59
Black	462	28.41
Deaths per year		
2010	126	7.61
2011	102	6.16
2012	110	6.65
2013	130	7.85
2014	166	10.03
2015	178	10.76
2016	236	14.26
2017	307	18.55
2018	300	18.13

* Missing data.

**Table 2:** Alzheimer’s mortality increase rate and 95% confidence interval between 2010 and 2018, Federal District, Brazil, 2010 to 2018 (*n* = 1655).

Group	Increment Rate (%)	95% Confidence Interval	Trend
Overall	33.40	18.89; 49.68	Increased
Sex			
Males	31.42	14.81; 50.44	Increased
Females	36.88	22.06; 53.51	Increased
Age (Years)			
45–59 years	-	-	-
60–69 years	6.09	−15.34; 29.82	Constant
70–79 years	14.66	−0.19; 31.74	Constant
≥80 years	23.81	10.30; 38.98	Increased

**Table 3:** Mean Alzheimer’s mortality rate according to administrative regions per 100,000 inhabitants, Federal District, Brazil, 2010 to 2018.

Administrative Region	Mortality Rate	95% Confidence Interval
Águas Claras	3.89	2.87–5.17
Brazlândia	3.41	2.05–5.33
Candangolândia	8.69	4.63–14.86
Ceilândia	4.52	3.88–5.25
Cruzeiro	15.41	11.20–20.70
Gama	8.48	6.96–10.25
Guará	11.63	9.73–13.81
Itapoã	0.95	0.12–2.24
Jardim Botânico	4.27	2.49–6.84
Lago Norte	18.80	14.45–24.05
Lago Sul	25.52	19.86–32.30
Núcleo Bandeirante	8.40	4.98–13.29
Paranoá	4.08	2.53–6.24
Park Way	11.04	6.92–16.71
Planaltina	3.01	2.24–3.98
Plano Piloto	15.27	13.59–17.12
Recanto das Emas	2.31	1.53–3.37
Riacho Fundo	3.87	3.68–5.41
Samambaia	3.14	2.41–4.03
Santa Maria	2.93	2.02–4.12
São Sebastião	2.20	1.30–3.48
Sobradinho	8.45	7.03–10.09
Sudoeste Octogonal	7.79	5.49–10.74
Taguatinga	9.78	8.41–11.33
Vicente Pires	5.95	4.19–8.20

## Data Availability

Data from this survey are available in a non-identifiable form at: https://datasus.saude.gov.br/ (accessed on 22 March 2023).

## Supplementary Materials

The following supporting information can be downloaded at: https://www.mdpi.com/article/10.3390/ijerph20186713/s1, Table S1: Rate of increase in mortality due to Alzheimer′s disease and respective confidence interval at 95%, according to the administrative region, Federal District, Brazil, 2010 to 2018 (N = 1655); Table S2: Mean mortality rate due to Alzheimer′s disease, according to gender and age group per 100,000 inhabitants, Federal District, Brazil, 2010 to 2018. Table S3: Mean Alzheimer′s mortality rate according to administrative regions per 100,000 inhabitants, Federal District, Brazil, 2010 to 2018.
