# Supplementary material for: Temporal Trends in Mortality from Alzheimer’s Disease in Federal District, Brazil: An Ecological Study (2010–2018)
Source: Int J Environ Res Public Health. 2023 Sep 5;20(18):6713. doi: 10.3390/ijerph20186713 (PMC10530803; doi:10.3390/ijerph20186713)
Supplement: Supplementary file 1 [file ijerph-20-06713-s001.zip › ijerph-2332223-supplementary.pdf]

SUPPLEMENTARY MATERIAL

Table S1 - Rate of increase in mortality due to Alzheimer's disease and respective confidence interval at 95%, according to the administrative region, Federal District, Brazil, 2010 to 2018 (N=1655).

ADMINISTRATIVE REGION	INCREMENT RATE (%)	CI 95%	TREND
Águas Claras	4.92	-38.20 ; 78.15	Constant
Brazlândia	24.00	-27.85 ; 113.16	Constant
Candangolândia	16.18	-5.64 ; 43.05	Constant
Ceilândia	37.54	25.47 ; 50.78	Increased
Cruzeiro	57.91	37.06 ; 81.93	Increased
Gama	43.92	17.88 ; 75.71	Increased
Guará	16.33	-13.44 ; 56.35	Constant
Itapoã	25.68	25.65 ; 25.71	Increased
Jardim Botânico	32.28	-31.03 ; 153.77	Constant
Lago Norte	43.08	4.28 ; 96.30	Increased
Lago Sul	53.02	10.68 ; 111.57	Increased
Núcleo Bandeirante	10.15	0.17 ; 21.12	Increased
Paranoá	18.79	-38.28 ; 128.64	Constant
Park Way	25.90	-9.52 ; 75.21	Constant
Planaltina	75.02	45.08 ; 111.13	Increased
Plano Piloto	26.02	16.12 ; 36.75	Increased
Recanto das Emas	48.32	32.37 ; 66.18	Increased
Riacho Fundo	49.48	14.34 ; 95.40	Increased
Samambaia	82.97	43.64 ; 133.07	Increased
Santa Maria	70.31	16.34 ; 149.31	Increased
São Sebastião	24.45	-13.94 ; 80.00	Constant
Sobradinho	44.47	8.42 ; 92.50	Increased
Sudoeste Octogonal	17.63	-27.59 ; 91.12	Constant
Taguatinga	21.39	-6.03 ; 56.82	Constant
Vicente Pires	50.35	23.25 ; 83.41	Increased

CI: Confidence interval.

Table S2 - Mean mortality rate due to Alzheimer's disease, according to gender and age group per 100,000 inhabitants, Federal District, Brazil, 2010 to 2018.

GROUP	MORTALITY RATE	95% CONFIDENCE INTERVAL
Overall	6.55	6.24-6.88
Males	4.86	4.48-5.27
Females	8.11	7.63-8.62
45-59 years	0.70	0.54-0.89
60-69 years	5.10	4.55-5.71
70-79 years	56.28	53.25-59.45

Table S3 – Mean Alzheimer's mortality rate according to administrative regions per 100,000 inhabitants, Federal District, Brazil, 2010 to 2018.

ADMINISTRATIVE REGION	MORTALITY RATE	95% CONFIDENCE INTERVAL

Águas Claras	3.89	2.87-5.17
Brazlândia	3.41	2.05-5.33
Candangolândia	8.69	4.63-14.86
Ceilândia	4.52	3.88-5.25
Cruzeiro	15.41	11.20-20.70
Gama	8.48	6.96-10.25
Guará	11.63	9.73-13.81
Itapoã	0.95	0.12-2.24
Jardim Botânico	4.27	2.49-6.84
Lago Norte	18.80	14.45-24.05
Lago Sul	25.52	19.86-32.30
Núcleo Bandeirante	8.40	4.98-13.29
Paranoá	4.08	2.53-6.24
Park Way	11.04	6.92-16.71
Planaltina	3.01	2.24-3.98
Plano Piloto	15.27	13.59-17.12
Recanto das Emas	2.31	1.53-3.37
Riacho Fundo	3.87	3.68-5.41
Samambaia	3.14	2.41-4.03
Santa Maria	2.93	2.02-4.12
São Sebastião	2.20	1.30-3.48
Sobradinho	8.45	7.03-10.09
Sudoeste Octogonal	7.79	5.49-10.74
Taguatinga	9.78	8.41-11.33
Vicente Pires	5.95	4.19-8.20

CI: Confidence interval.
